# Luminescent Mesoporous Silica Nanohybrid Based on Drug Derivative Terbium Complex

**DOI:** 10.3390/ma12060933

**Published:** 2019-03-21

**Authors:** Fernando E. Maturi, Rafael M. Sábio, Robson R. Silva, Marcelo G. Lahoud, Andréia B. Meneguin, Gustavo T. Valente, Raphael A. Caface, Ilaiáli S. Leite, Natalia M. Inada, Sidney J. L. Ribeiro

**Affiliations:** 1 Institute of Chemistry, São Paulo State University-UNESP, 14800-060 Araraquara-SP, Brazil; fernando.maturi@unesp.br (F.E.M.); marcelo_lahoud@hotmail.com (M.G.L.); sidney.jl.ribeiro@unesp.br (S.J.L.R.); 2 São Carlos Institute of Physics, University of São Paulo-USP, 13560-970 São Carlos-SP, Brazil; robsilva31@usp.br (R.R.S.); abagliottim@hotmail.com (A.B.M.); gtvfisica@gmail.com (G.T.V.); raphaelcaface@gmail.com (R.A.C.); ilaiali.leite@usp.br (I.S.L.); nataliainada@gmail.com (N.M.I.)

**Keywords:** terbium, ketoprofen, mesoporous silica nanoparticles, two-photon, luminescent nanohybrid

## Abstract

Mesoporous silica nanoparticles prepared by organic template-driven synthesis have been successfully explored as carriers of the drug-derivate green luminescent complex of terbium (III) with the nonsteroidal anti-inflammatory drug ketoprofen. The terbium (III) complex was synthesized by reacting ketoprofen sodium salt with terbium (III) chloride, which was further adsorbed onto the surface of mesoporous nanoparticles with a mean particle size of 47 ± 4 nm and pore size of 11 nm. The incorporation of the complex into mesoporous silica nanoparticles was tracked by the decrease in the surface area and pore size of the nanoparticles, and successfully demonstrated by substantial changes in the adsorption isotherms and thermal and vibrational spectroscopy results. The cytotoxicity assay and confocal microscopy have shown that the novel luminescent nanohybrid presents high cell viability and the characteristic terbium (III) emission can be assessed through two-photon excitation, which paves the way for bioimaging applications in nanomedicine.

## 1. Introduction

Trivalent lanthanide ions (Ln^3+^) have been extensively studied due to their unique optical properties, such as long emission lifetimes, high quantum yields, and sharp emission bands ranging from the ultraviolet (UV) to near-infrared (NIR) spectral regions [1,2,3,4,5,6]. These properties are suitable for a myriad of applications, including bioimaging [7,8], luminescent thermometers [9], light-emitting diodes (LEDs) [10], and telecommunication [11]. However, the forbidden intra-configurational 4f-4f transitions of the Ln^3+^ present small molar absorption coefficients (ε < 10 L mol^−1^ cm^−1^), leading to low emission intensities [12]. To avoid this issue, Ln^3+^ are usually coordinated with organic chromophores to obtain complexes that can harvest the excitation light and transfer it to the metal center, sensitizing the emission through the so-called “antenna effect” [13].

Although they have good optical properties, there are still limitations in the biological applications of Ln^3+^ complexes. The sensitization of lanthanide ions, such as Eu^3+^ and Tb^3+^, occurs through UV light excitation, resulting in scattering and absorption of these wavelengths by the biological medium, beyond the low solubility in aqueous media and phototoxicity [14,15,16]. The design of Ln^3+^ complexes using ligands with two-photon induced emission can be used to overcome the photodamage risks to biological tissues [17]. The two-photon (2P) excitation is a nonlinear process relying on the absorption of two photons where the excited state is accessed by using photons of half the energy of the photon used in the one-photon (1P) excitation [18]. Ligands with broad excitation bands around 350 nm are suitable for using NIR as the excitation source, and 2P excitation of lanthanide complexes have been studied in the 700–1000 nm range, within the spectral region where biological media present higher transparency [12,14,17,19,20]. 

The main key to achieving light-emitting functional materials for biological applications is a meticulous choice of the ligand used in the Ln^3+^ complex synthesis. Non-steroidal anti-inflammatory drugs (NSAID) such as ketoprofen and ibuprofen are structurally similar to organic chromophores, allowing the Ln^3+^ sensitization through both 1P and 2P excitation, besides the low cytotoxicity, paving the way for pharmacological, diagnostic, and therapeutic applications [21,22,23,24]. The poor water solubility, mechanical properties, and thermal stability are circumvented by incorporating the drug derivative Ln^3+^ complexes into solid inorganic matrices, which have been used for the development of new optical functional materials based on Ln^3+^ complexes [25,26,27].

Mesoporous silica nanoparticles (MSNs) arise among the variety of existing host matrices. The MSN structure allows for the incorporation of large amounts of complex due to their high surface area (>900 m^2^ g^−1^), large pore volume (>0.9 cm^3^ g^−1^), and narrow pore size distribution (2 to 10 nm). They also present good biocompatibility, water stability, and free silanol groups available for functionalization [28,29,30,31]. The incorporation of complexes into MSNs is usually made by grafting them onto a silicate surface or trapping them within the porous channels [32,33].

The preparation of a drug derivative Ln^3+^ complex followed by its incorporation into MSN pores offers an alternative approach for the development of water-dispersible nanomaterials with low cytotoxicity presenting 2P-induced emission. This is interesting mainly in the field of nanomedicine [4,34]. In this way, we herein report the development of a new green-emitting nanohybrid based on the incorporation of a Tb^3+^ ketoprofen complex into MSNs, presenting its 1P and 2P studies and cytotoxicity evaluation.

## 2. Materials and Methods

### 2.1. Materials

Ketoprofen sodium salt (98%), terbium (III) chloride hexahydrate (99.9%), anhydrous ethyl acetate (99.8%), cetyltrimethylammonium bromide (CTAB, 99%), octane (98%), styrene monomer (99%), L-lysine (98%), tetraethylorthosilicate (TEOS, 99%), 2,2′-azobis(2-methylpropionamidine) dihydrochloride (AIBA, 97%), and 3-[4,5-dimethylthiazol-2-yl]-2,5-diphenyltetrazolium bromide (MTT) were purchased from Sigma-Aldrich (St. Louis, MO, USA). Phenol-free Dulbecco’s Modified Eagle’s Medium (DMEM) and fetal bovine serum (FBS) were supplied by Cultilab (Campinas, Brazil). A neonatal human dermal fibroblast (HDFn) cell line was purchased from Thermo Fisher Scientific (Waltham, MA, USA).

### 2.2. Synthesis

Tb^3+^ Complex. The synthesis of [Tb(ket)_3_]∙0.5H_2_O complex (TbKet) was based on previous work [22]. The pH of an aqueous solution of ketoprofen sodium salt (0.1 mol L^−1^) was adjusted to 8.0 by the addition of an aqueous solution of sodium hydroxide (0.1 mol L^−1^). Under continuous stirring, the ketoprofen solution was added dropwise to a terbium chloride solution (0.1 mol L^−1^) until precipitation. The resulting solid was washed with distilled water, filtered, dried on Whatman no. 42 paper filter in order to eliminate chloride ions, and kept in a desiccator under vacuum over anhydrous calcium chloride.

Mesoporous Silica Nanoparticles. MSNs were prepared using an organic template-driven synthesis as previously reported [35]. In a three-neck round-bottom flask, CTAB (0.1 g) was dissolved in 30 mL of water. After 30 min of mixing at 60 °C, octane, styrene monomer, L-lysine, TEOS, and AIBA were added to the flask. The reaction was carried out for 3 h at 60 °C under N_2_ atmosphere. After heating, the suspension was cooled to room temperature for 12 h and centrifuged (15,000 rpm) twice using ethanol to wash the nanoparticles. The organic template was removed by heat treatment at 600 °C under atmospheric conditions.

Nanohybrid. For the preparation of TbKet containing MSN nanohybrid (SiTb), 200 mg of MSNs was previously dried in a desiccator under vacuum for 4 h. The MSN were added into a solution of 100 mg of TbKet complex dissolved in 20 mL of ethyl acetate and stirred for 3 days. The final product was centrifuged and washed three times with ethyl acetate.

### 2.3. Instrumentation

The transmission electron microscopy (TEM) and scanning electron microscopy (SEM) images were acquired on a Philips CM20 (200 kV) microscope (Amsterdam, The Netherlands) and a JEOL 7500F (2 kV) equipment (Akishima, Tokyo), respectively. Infrared vibrational (FTIR) spectra were registered on a Nicolet spectrophotometer model Impact 400 FT-SX (Thermo Fisher Scientific Waltham, MA, USA) in the region from 4000 to 400 cm^−1^, using 64 scans with a resolution of 4 cm^−1^ in the attenuated total reflectance mode (ATR). The thermogravimetric analysis (TGA) curves were obtained on a TA Instruments equipment model SDT 2960 simultaneous differential thermal analysis-thermal gravimetric analysis (DTA-TGA, TA Instruments, New Castle, DE, USA) and the samples were heated at 10 °C min^−1^ from 25 °C to 750 °C in dynamic air atmosphere (flow rate = 100 mL min^−1^). Elemental analysis (EA) of C and H was performed on a Fisons Element Analyzer model EA1110 CHNS-O (Fisons, Ipswich, UK). The incorporation efficiency was calculated in mmol of complex per gram of silica using EA and TGA [36,37].

The excitation and emission spectra of the samples in solid-state at room temperature were registered on a Horiba Jobin Yvon spectrofluorimeter model Fluorolog FL3-222 (Piscataway, NJ, USA) equipped with a Hamamatsu R928 photomultiplier (Hamamatsu, Japan) and Xe lamp (450 W), and using the front face excitation mode. Emission decay measurements were performed on the same equipment by using a pulsed Xe lamp (3 μs bandwidth). Nitrogen adsorption-desorption isotherms were measured on a Micromeritics ASAP 2020 Surface Area & Porosity Analyzer (Micromeritics, Norcross, GA, USA), and the experiments were carried out on samples degassed at 200 °C under vacuum for 24 h. The pore size and total pore volume of the samples were estimated from the adsorption average pore width and the amount of N_2_ adsorbed at P/P_0_ = 0.99, respectively.

Laser scanning confocal microscopy (LSCM) was used to verify the viability of 2P excitation of the SiTb nanohybrid. A Zeiss LSM 780 laser scanning confocal microscope (Zeiss, Jena, Germany) with coherent Chameleon laser (Ti: sapphire) was used in the experiments at 700 nm as the 2P excitation source. An aqueous suspension of the SiTb nanohybrid (100 µg mL^−1^) was observed with a C-Apochromat objective lens (40×, numerical aperture 1.0 and water immersion) with a laser output power of 36 mW on the opposite side of the coverslip. A 200 nm spatial resolution was used for numerical aperture and excitation wavelength. The obtained confocal image consists of a 1024 × 1024 pixel area (212.34 μm × 212.34 μm) and each pixel corresponds to an emission spectrum of the SiTb nanohybrid.

Cytotoxicity was evaluated by the MTT assay using neonatal human dermal fibroblast (HDFn) cell lines. Cells were cultivated in DMEM supplemented with 10% (v/v) FBS in a humidified incubator at 37 °C under 5% CO_2_ atmosphere. To assess SiTb effects on cellular viability of the healthy cells, monolayer cultures were exposed to the nanohybrid. The 96-well plates were seeded with DMEM supplemented with 10% FBS containing 5 × 10^4^ cells mL^−1^ 24 h prior to exposure to the samples. Cells were inoculated with SiTb aqueous solutions ranging from 12.5 to 100 µg mL^−1^, prepared immediately before its use (using an ultrasonic bath to redisperse the nanohybrid and avoid aggregation) in DMEM supplemented with 10% FBS, and incubated for 24 and 48 h in a humidified incubator at 37 °C under 5% CO_2_ atmosphere. Cell viability was obtained indirectly by measuring the MTT absorbance at 570 nm with the microplate spectrophotometer MultiskanTM Go (Thermo Fisher Scientific, Waltham, MA, USA) and viability percentages were calculated based on the absorbance values of the control group (cells that were not exposed to the nanohybrid). Statistical analysis of the data was performed using the Analysis of Variance (ANOVA) followed by Tukey’s multiple comparisons. The analysis was performed 24 h after incubation in the dark and in the presence of four different prepared SiTb concentrations.

## 3. Results

**Figure 1:**
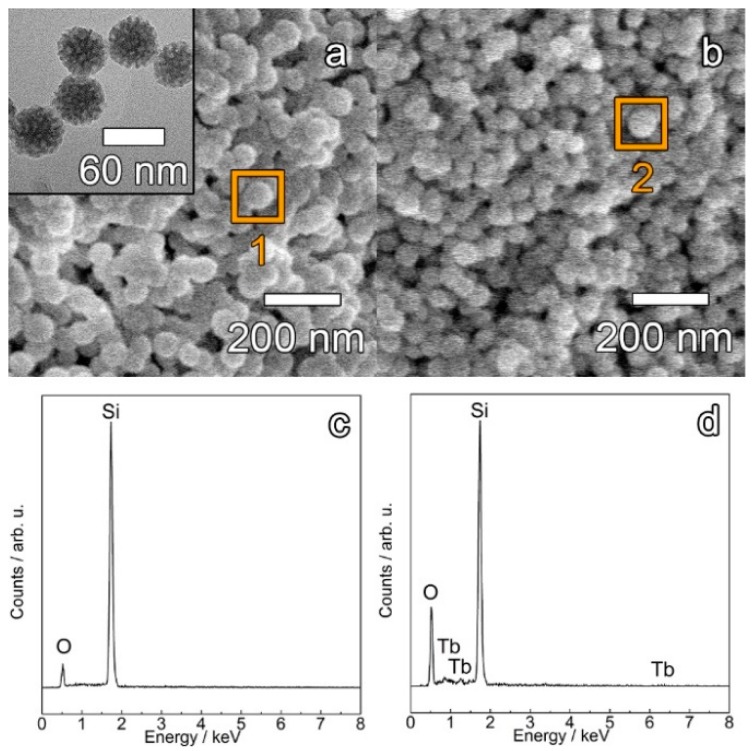
SEM images of pristine MSNs (**a**) and the SiTb nanohybrid (**b**). The EDX spectra of pristine MSNs (**c**) and the SiTb nanohybrid (**d**) were obtained from regions 1 and 2 highlighted in panels (**a**) and (**b**), respectively. The inset in (**a**) shows the TEM image of pristine MSNs.

The obtained MSNs displayed a spherical shape with a mean particle size of 47 ± 4 nm, as shown in Figure 1. The height contrast displayed in the TEM image of pristine MSN highlights the large porous surface (pore channels appear in bright contrast). The incorporation of the TbKet complex did not substantially change the shape or average size of the nanoparticles as evidenced in Figure 1a,b. As expected, the energy-dispersive X-ray (EDX) spectrum acquired from a delimited region of pristine MSN sample (Figure 1c) shows only two peaks, corresponding to Si and O signals of the MSN matrix. Indeed, the EDX spectrum of the nanohybrids (Figure 1d) unveils the presence of three weak peaks corresponding to the Tb signal and confirming the incorporation of the TbKet complex in the MSNs.

**Figure 2:**
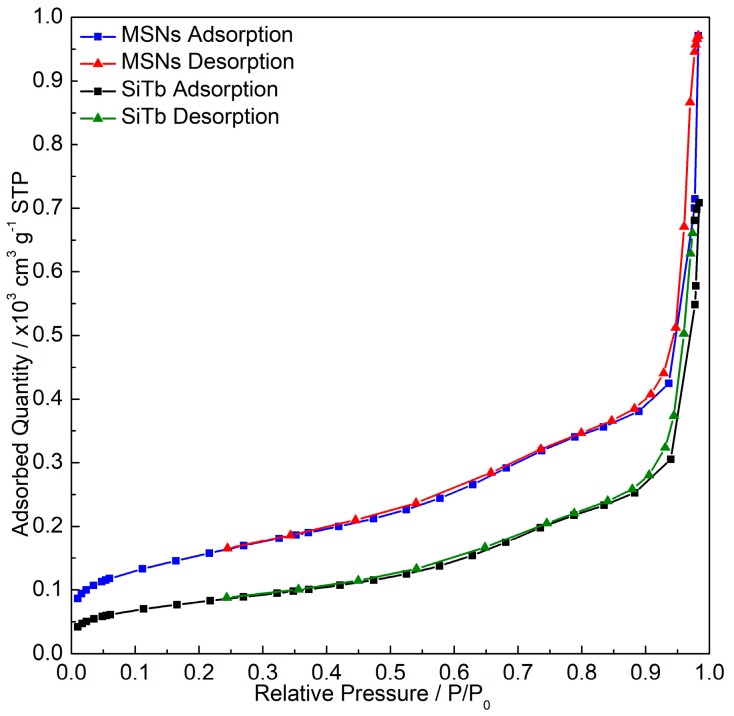
N_2_ adsorption-desorption isotherm of MSNs and SiTb nanohybrid.

The N_2_ adsorption-desorption isotherm of the samples in Figure 2 reveals that the obtained MSNs present a type IV isotherm with a type H1 hysteresis loop, which are characteristics of mesoporous materials according to the international union of pure and applied chemistry (IUPAC) classification [38]. The pore size of pristine MSNs estimated from Figure 2 is around 11 nm. The surface area decreased from 542 m^2^ g^−1^ in MSNs to 286 m^2^ g^−1^ in the SiTb nanohybrid, suggesting that the incorporation of TbKet took place. The total pore volume also decreased from 1.50 in the MSNs to 1.10 cm^3^ g^−1^ in the SiTb, confirming that the incorporation of the TbKet complex into the MSN pores was achieved.

**Figure 3:**
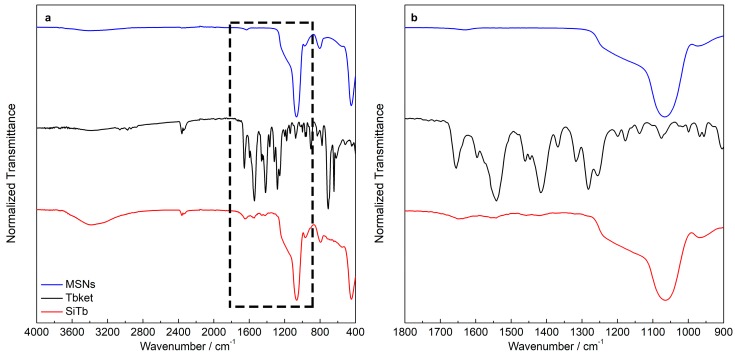
FTIR of pristine MSNs, TbKet complex, and SiTb nanohybrid (**a**). Amplified 1800–900 cm^−1^ region of samples (**b**).

Figure 3 presents the FTIR spectra of MSNs and SiTb where the ν(Si–O–Si) and ν(Si–OH) stretching signals are seen at 1066 and 969 cm^−1^, respectively [39]. The bands found at 1655 cm^−1^ (ν(C=O)), 1541 cm^−1^ (antisymmetric ν(COO^−^)), and 1415 cm^−1^ (symmetric ν(COO^−^)) for TbKet were also found in the SiTb nanohybrid [22]. Additionally, no shifts of the TbKet complex vibrational bands were observed after the incorporation in MSNs, suggesting that TbKet was non-covalently bonded to the MSN surface.

**Figure 4:**
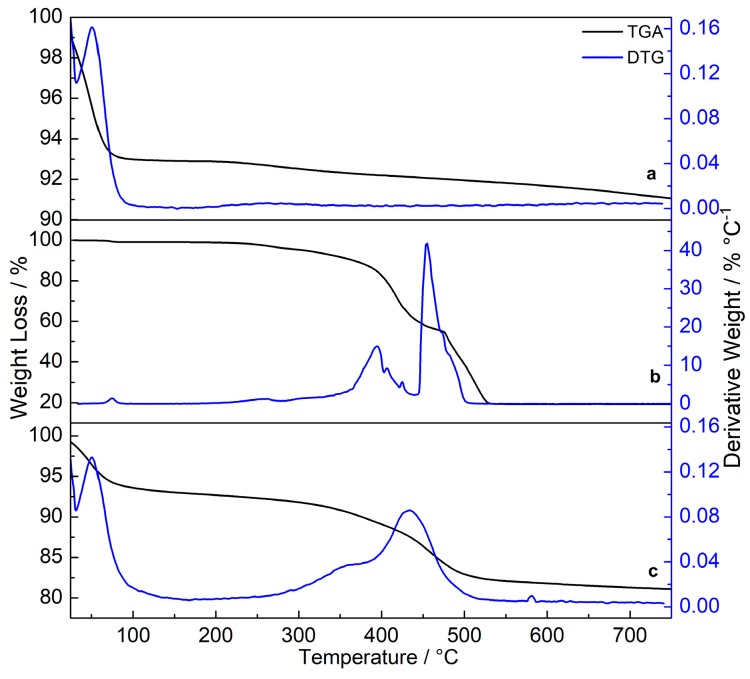
Thermogravimetric analysis (TGA) and DTG curves of pristine MSNs (**a**), TbKet complex (**b**) and SiTb nanohybrid (**c**).

Two main events are present in the thermogravimetric curve of the MSNs in Figure 4a. The first, from 25 to 150 °C (6.88%), corresponds to the removal of solvent molecules adsorbed in the silica matrix while the second between 150 and 750 °C (2.03%) is ascribed to dehydroxylation (loss of OH groups) of MSNs. Furthermore, the lack of mass losses and stretching signals from the organic material in TGA and FTIR results, respectively, confirm the absence of organic residues after the heat treatment of pristine MSNs [36,40]. The TGA curve obtained from the TbKet complex (Figure 4b) shows two events where the mass losses are attributed to the dehydration (0.92%) and the decomposition of the organic ligands (79.36%) [41]. The TGA results obtained from the SiTb nanohybrid (Figure 4c) are in accordance with pristine MSNs and TbKet complex results. The first event from 25 to 150 °C is ascribed to the dehydration (6.97%) of the nanohybrid. The second one from 200 to 600 °C is assigned to the oxidative decomposition of the organic ligands (10.76%). From 600 to 750 °C (Figure 4c), a slight mass loss is observed and it is related to the dehydroxylation of the SiTb nanohybrid.

The mass loss from the SiTb nanohybrid (ascribed to the organic ligands) was used to calculate the incorporation efficiency, expressed in mmol of complex per gram of silica [36]. The incorporation efficiency of the TbKet complex into the MSNs obtained from the TGA results was 0.12 mmol g^−1^ of silica, which is in good agreement with the incorporation efficiency calculated from elemental analysis data (0.12 mmol g^−1^) [29,36,37,42]. The TGA and elemental analysis measurements confirm the incorporation of the TbKet complex inside the pores of pristine MSNs, corroborating the isotherm curves and FTIR results. Additionally, the OH groups present on the surface of the SiTb nanohybrid are still available for further functionalization.

**Figure 5:**
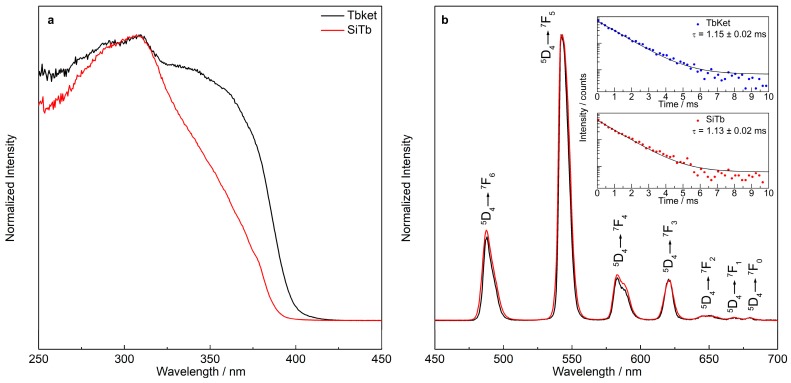
Excitation spectra monitoring emission at 542 nm (**a**) and emission spectra under 310 nm excitation of the TbKet and SiTb nanohybrid (**b**). The inset in (**b**) shows the emission decay curve of the TbKet complex and SiTb nanohybrid.

Figure 5 displays the excitation and emission spectra of the TbKet complex and SiTb nanohybrid, where the characteristic bands of the TbKet complex are seen. The broad band between 250 and 400 nm in the excitation spectra (Figure 5a) arises from π→π* and *n*→π* intra-ligand transitions [22]. The complex displays a high absorption in the UV region, leading to a broader band than that observed in the nanohybrid where this effect is minimized due to the dilution of the complex in the MSN host [43,44]. The same emission profile is seen for the emission spectra of both samples (Figure 5b) and the following Tb^3+^ transitions from ^5^D_4_ to ^7^F_J_ (J = 0–6) levels in the SiTb nanohybrid were observed: ^5^D_4_→^7^F_6_ (487 nm), ^5^D_4_→^7^F_5_ (542 nm), ^5^D_4_→^7^F_4_ (583 nm), ^5^D_4_→^7^F_3_ (620 nm), ^5^D_4_→^7^F_2_ (650 nm), ^5^D_4_→^7^F_1_ (668 nm), and ^5^D_4_→^7^F_0_ (679 nm).

The experimental lifetime (τ) of the ^5^D_4_→^7^F_5_ transition for the SiTb nanohybrid was calculated monitoring the emission at 542 nm under excitation at 310 nm and by fitting the luminescence decay curve with a first-order single exponential decay function, where τ = 1.13 ± 0.02 ms was obtained. The lifetime of the SiTb nanohybrid is in accordance with the lifetime obtained for the TbKet complex (τ = 1.15 ± 0.02 ms). It is worth pointing out that the emission bands and lifetime of Tb^3+^ are preserved after the incorporation of the TbKet complex into the MSN.

**Figure 6:**
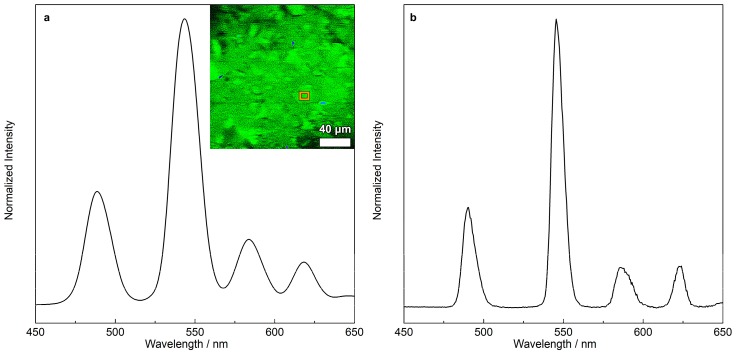
LSCM emission spectrum under two-photon excitation at 700 nm (**a**) and emission spectrum under one-photon excitation at 310 nm (**b**). The inset panel in (**a**) presents the false-colored spectral confocal image of the SiTb nanohybrid with the analyzed region highlighted in orange.

The LSCM image of Figure 6 shows the emission of an aqueous suspension of the SiTb nanohybrid (100 µg mL^−1^). The emission spectrum under 2P excitation (Figure 6a) presents a similar spectral lineshape for the SiTb nanohybrid excited by 1P (Figure 6b). In the 2P case, 700 nm was used as the excitation wavelength because its 1P corresponding excitation wavelength (350 nm) falls within the broad excitation band of SiTb, allowing the excitation in the NIR spectral region [19]. After the absorption of two photons by the ketoprofen ligand, its triplet state is accessed and then the energy is transferred to the ^5^D_4_ emitting level of Tb^3+^ via an intramolecular energy transfer, where the emission occurs due to the ^5^D_4_→^7^F_J_ (J = 0–4) transitions [16]. Consequently, the green pseudocolor in the confocal image comes from the strong luminescence resulting from the ^5^D_4_→^7^F_5_ transition of Tb^3+^ (Figure 6a, inset).

**Figure 7:**
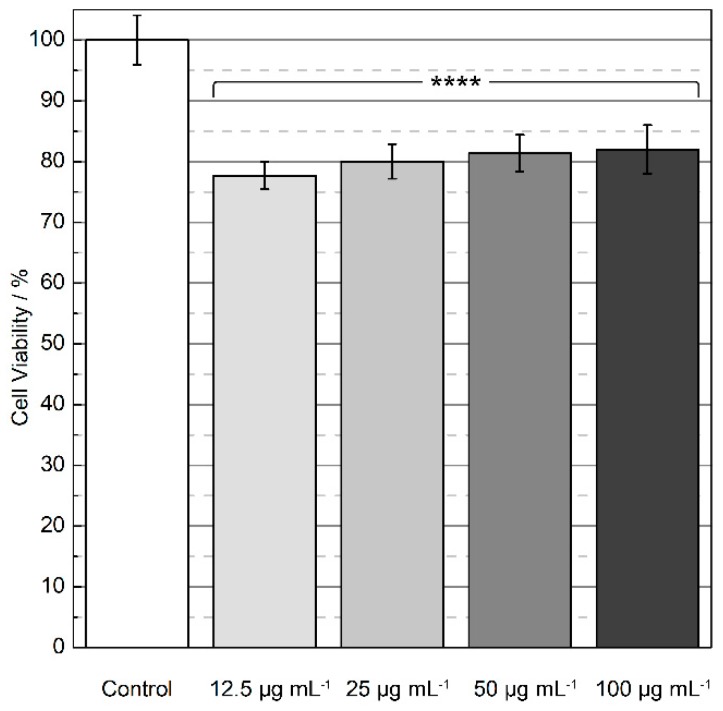
Cell viability (percentual values) as a function of the SiTb nanohybrid concentration (µg mL^−1^) was assessed by the MTT assay. Each value represents the mean ± standard deviation (n = 10, with **** indicating a statistically significant difference between the groups when compared to the control group, according to Tukey’s multiple comparisons (p < 0.0001)).

Cell viability was evaluated by the MTT assay and was performed to analyze the cytotoxicity of the SiTb nanohybrid at different concentrations. The related data are shown in Figure 7. A neonatal fibroblast cell line was tested to determine whether the compound has cytotoxic properties. Slight cytotoxicity (20%) was observed in all conditions tested (with p < 0.0001 when groups that were exposed to the nanoparticles were compared to cells that were not), which suggests that the SiTb is biocompatible for concentrations up to 100 µg mL^−1^, the highest concentration evaluated in the present study.

The same level of cytotoxicity for nanoparticles containing Tb^3+^ was verified by Shang and collaborators with calcium phosphate nanoparticles doped with this lanthanide [45]. When human glioma cells were co-cultured for 48 h with 5–150 µg mL^−1^ solutions of the nanoparticles, viability values remained over 80%. In a similar way, cells derived from cervical cancer (HeLa cells) were exposed to Eu-linked mesoporous silica nanospheres for 24 h [46]. Samples also presented viability values close to 100% for concentrations ranging from 3.1–800 µg mL^−1^. HeLa cells, alongside with human lung adenocarcinoma cells, were assessed as the in vitro model for cytotoxicity of the Tb^3+^ inorganic-organic mesoporous hybrid nanospheres reported by Zhou et al. [47]. Concentrations from 5–100 µg mL^−1^ were incubated with both cell lines for 24 h, resulting in cell viability above 90% up to 50 µg mL^−1^ and close to 70% when 100 µg mL^−1^ was evaluated. Ge et al. synthesized mesoporous upconversion nanoparticles with a Tb^3+^ complex and tested 12.5–400 µg mL^−1^ of the nanoparticles in murine RAW 264.7 macrophages [48]. After 24 h of exposition, the immune cells presented viability values ≥95% for all the groups. Gálico et al. have also studied the TbKet complex, including their influence on peripheral viability of blood mononuclear cells [22]. Solutions of approximately 15, 30, and 61 mg mL^−1^ of the complex were kept in contact with the cells for 96 h, and displayed no cytotoxicity. Although the absence of acute cytotoxicity for Tb^3+^ complexes and ketoprofen has been previously reported in the literature, the use of the herein prepared SiTb nanohybrid paves the way for new applications in biomedicine due to the feasible way of incorporating the TbKet complex into MSNs without the need of grafting.

## 4. Conclusions

We have presented here the preparation of a green-emitting nanohybrid obtained through the incorporation of a terbium (III) ketoprofen complex into mesoporous silica nanoparticles. The electron microscopy images showed that the obtained MSNs present a mean size of 47 nm and there were no changes in the morphology after the incorporation of the TbKet complex. An incorporation efficiency of 0.12 mmol of TbKet per gram of MSNs was found by TGA and EA, highlighting the feasibility of using the porous structure of MSNs for incorporating considerable amounts of the complex without the need of grafting or functionalization. The SiTb nanohybrid presents the characteristic Tb^3+^ ^5^D_4_→^7^F_J_ emission lines, which can be seen under either 1P or 2P excitation. The cell viability of approximately 80% found for the four concentrations of the nanohybrid studied (12.5–100 µg mL^−1^) suggests good biocompatibility. Therefore, the obtained SiTb is suitable for bioimaging purposes where 2P excitation can be used to reduce the photodamage to biological tissues.
